# Supplementary material for: Scalable gradients enable Hamiltonian Monte Carlo sampling for phylodynamic inference under episodic birth-death-sampling models
Source: bioRxiv. 2023 Nov 2:2023.10.31.564882. Preprint. [Version 1] doi: 10.1101/2023.10.31.564882 (PMC10634968; doi:10.1101/2023.10.31.564882)
Supplement: 1 [file NIHPP2023.10.31.564882v1-supplement-1.pdf]

bioRxiv preprint doi: https://doi.org/10.1101/2023.10.31.564882; this version posted November 2, 2023. The copyright holder for this preprint (which was not certified by peer review) is the author/funder, who has granted bioRxiv a license to display the preprint in perpetuity. It is made available under aCC-BY 4.0 International license.

Supplementary Material

S1 Likelihood Derivation

S1.1 Formulas for Likelihood Related Functions

$$A_k = \sqrt{(\lambda_k - \mu_k - \psi_k)^2 + 4\lambda_k\psi_k}, \tag{19}$$

$$B_k = \frac{(1 - 2\,(1 - \rho_k)\,p_{k-1}(t_{k-1}))\,\lambda_k + \mu_k + \psi_k}{A_k} \tag{20}$$

$$p_k(t) = \frac{\lambda_k + \mu_k + \psi_k - A_k \frac{e^{A_k(t-t_{k-1})}(1+B_k)-(1-B_k)}{e^{A_k(t-t_{i-1})}(1+B_k)+(1-B_k)}}{2\lambda_k} \tag{21}$$

$$q_k(t) = \frac{4e^{A_k(t-t_{k-1})}}{\left(e^{A_k(t-t_{k-1})}(1 + B_k) + (1 - B_k)\right)^2} \tag{22}$$

$$g_1 = e^{A_k(t-t_{k-1})} \cdot (1 + B_k) + (1 - B_k) \tag{23}$$

$$g_2 = A_k(1 - \frac{2(1 - B_k)}{g_1}) \tag{24}$$

$$g_3 = 1 - 2\,(1 - \rho_k))P_{k-1}(t_{k-1}) \tag{25}$$

bioRxiv preprint doi: https://doi.org/10.1101/2023.10.31.564882; this version posted November 2, 2023. The copyright holder for this preprint (which was not certified by peer review) is the author/funder, who has granted bioRxiv a license to display the preprint in perpetuity. It is made available under aCC-BY 4.0 International license.

S1.2 Implementation Algorithm:

Detailed algorithm for likelihood calculation is shown below based on the equations listed in Section 2.2 of the main text and from the section above.

Algorithm 1: Likelihood Calculation

Initialize: $p_0(t_0) = 1$
for $k = 0, \ldots, K-1$ **do**
    `/* Intermediate quantities */`
    Load the value of $p_k(t_k)$
    Calculate A_{k+1}, B_{k+1} via Equation (19), (20)
    for $j = 0, \ldots, m_{k+1} - 1$ **do**
        Calculate $q_{k+1}(s_{j+1})$ via Equation (22)
        if s_{j+1} is a serial sampling event **then**
            Calculate $p_{k+1}(s_{j+1})$ via Equation (21)
        end
        if $j \geq 1$ **then**
            Calculate $I_k(E_j)$ via Equation (2)
        end
    end
    Calculate and store $p_{k+1}(t_{k+1})$ via Equation (21)
end
`/* Likelihood */`
Calculate $\mathbb{P}[\mathcal{T} \mid \boldsymbol{\lambda}, \boldsymbol{\mu}, \boldsymbol{\psi}, \boldsymbol{\rho}, \boldsymbol{r}, \boldsymbol{t}]$ via Equation (1)

---

bioRxiv preprint doi: https://doi.org/10.1101/2023.10.31.564882; this version posted November 2, 2023. The copyright holder for this preprint (which was not certified by peer review) is the author/funder, who has granted bioRxiv a license to display the preprint in perpetuity. It is made available under aCC-BY 4.0 International license.

S2 Gradient Derivation

S2.1 For $\frac{\partial \mathbb{P}_k(j)}{\partial \theta_k}$:

$$
\begin{aligned}
\frac{\partial q_k(t)}{\partial \theta_k} &= \frac{8e^{A_k(t-t_{k-1})}((t-t_{k-1})\frac{\partial A_k}{\partial \theta_k}(\frac{1}{2}\cdot g_1 - e^{A_k(t-t_{k-1})}\cdot(1+B_k))}{g_1^3} \\
&\quad - \frac{\frac{\partial B_k}{\partial \theta_k}(e^{A_k(t-t_{k-1})}-1))}{g_1^3}
\end{aligned} \tag{26}
$$

$$
\frac{\partial A_k}{\partial \theta_k} = \begin{cases} \frac{\lambda_k-\mu_k+\psi_k}{A_k}, & \text{If } \theta=\lambda \\ \frac{-\lambda_k+\mu_k+\psi_k}{A_k}, & \text{If } \theta=\mu \\ \frac{\lambda_k+\mu_k+\psi_k}{A_k}, & \text{If } \theta=\psi \\ 0, & \text{If } \theta=\rho \end{cases} \tag{27}
$$

$$
\frac{\partial B_k}{\partial \theta_k} = \begin{cases} \frac{2\lambda_k p_{k-1}(t_{k-1})}{A_k}, & \text{If } \theta=\rho \\ \frac{\partial B_k}{\partial \theta_k} = \frac{A_k\cdot\text{temp} - \frac{\partial A_k}{\partial \theta_k}\cdot(g_3\cdot\lambda_k+\mu_k+\psi_k)}{A_k^2}, & \text{Otherwise} \end{cases} \tag{28}
$$

$$
\frac{\partial p_k(t)}{\partial \theta_k} = \begin{cases} \frac{1}{2\lambda_k^2}(-\mu_k-\psi_k-\lambda_k\frac{\partial g_2}{\partial \lambda_k}+g_2), & \text{If } \theta=\lambda \\ -\frac{A_k}{\lambda_k}\frac{\left((1-B_k)(e^{A_k(t-t_{k-1})}-1)+g_1\right)\frac{\partial B_k}{\partial \rho_k}}{g_1^2}, & \text{If } \theta=\rho \\ \frac{1}{2\theta_k}(1-\frac{\partial g_2}{\partial \theta_k}), & \text{Otherwise} \end{cases} \tag{29}
$$

$$
\frac{\partial Q_k(s_{j+1},s_j)}{\partial \theta_k} = \frac{1}{q_k(s_{j+1})}\frac{\partial q_k(s_{j+1})}{\partial \theta_k} - \frac{1}{q_k(s_j)}\frac{\partial q_k(s_j)}{\partial \theta_k} \tag{30}
$$

$$
\begin{aligned}
\frac{\partial g_2}{\partial \theta_k} &= \frac{dA_k}{d\theta_k} - \frac{2}{g_1^2}\cdot\Bigg(g_1\left\{\frac{dA_k}{d\theta_k}(1-B_k) - \frac{dB_k}{d\theta_k}\cdot A_k\right\} \\
&\quad - \left(e^{A_k(t-t_{k-1})}\frac{\partial A_k}{\partial \theta_k}(1+B_k)\cdot(t-t_{k-1}) + (e^{A_k(t-t_{k-1})}-1)\frac{\partial B_k}{\partial \theta_k}\right)\cdot A_k(1-B_k)\Bigg)
\end{aligned} \tag{31}
$$

bioRxiv preprint doi: https://doi.org/10.1101/2023.10.31.564882; this version posted November 2, 2023. The copyright holder for this preprint (which was not certified by peer review) is the author/funder, who has granted bioRxiv a license to display the preprint in perpetuity. It is made available under aCC-BY 4.0 International license.

S2.2 For $\frac{\partial \mathbb{P}_k(j)}{\partial \theta_{k-i}}$ (i is an integer smaller than k):

$$\frac{\partial q_k(t)}{\partial \theta_{k-i}} = -\frac{8e^{A_k(t-t_{k-1})}\frac{\partial B_k}{\partial \theta_{k-i}}(e^{A_k(t-t_{k-1})}-1)}{g_1^3} \tag{32}$$

$$\frac{\partial B_k}{\partial \theta_{k-i}} = \frac{\partial B_k}{\partial p_{k-1}(t_{k-1})} \cdot \frac{\partial p_{k-1}(t_{k-1})}{\partial \theta_{k-i}} = \frac{-2(1-\rho_k)\lambda_k}{A_k}\frac{\partial p_{k-1}(t_{k-1})}{\partial \theta_{k-i}} \tag{33}$$

$$\frac{\partial p_k(t)}{\partial \theta_{k-i}} = -\frac{A_k}{\lambda_k}\frac{\left((1-B_k)(e^{A_k(t-t_{k-1})}-1)+g_1\right)\frac{\partial B_k}{\partial \theta_{k-i}}}{g_1^2} \tag{34}$$

$$\frac{\partial Q_k(s_{j+1},s_j)}{\partial \theta_{k-i}} = \frac{1}{q_k(s_{j+1})}\frac{\partial q_k(s_{j+1})}{\partial \theta_{k-i}} - \frac{1}{q_k(s_j)}\frac{\partial q_k(s_j)}{\partial \theta_{k-i}} \tag{35}$$

bioRxiv preprint doi: https://doi.org/10.1101/2023.10.31.564882; this version posted November 2, 2023. The copyright holder for this preprint (which was not certified by peer review) is the author/funder, who has granted bioRxiv a license to display the preprint in perpetuity. It is made available under aCC-BY 4.0 International license.

S2.3 Implementation Algorithm:

We implement a recursive algorithm to compute the necessary gradient of the log-likelihood within our rate parameter space. Intermediate quantities are stored in between epochs to alleviate computational burden. Detailed algorithm is shown below based on the equations listed in 2.5 and previous sections in the supplement.

Algorithm 2: Gradient Calculation

Initialize: $p_0(t_0) = 1$
for $k = 0, \dots, K-1$ **do**
  /* Intermediate quantities */
  if $k == 0$ **then**
    Calculate $\frac{\partial A_1}{\partial \theta_1}$, $\frac{\partial B_1}{\partial \theta_1}$ using $p_0(t_0)$ via Equation (27), (28)
  end
  else if $k \geq 1$ **then**
    Load the values of $\{\frac{\partial p_k(t_k)}{\partial \theta_i}\}_{i=1}^{k}$
    Calculate $\frac{\partial A_{k+1}}{\partial \theta_{k+1}}$, $\{\frac{\partial B_{k+1}}{\partial \theta_i}\}_{i=1}^{k+1}$ using $\{\frac{\partial p_k(t_k)}{\partial \theta_i}\}_{i=1}^{k}$ via Equation (27), (28), (33)
  end
  Calculate and store $\{\frac{\partial p_{k+1}(t_{k+1})}{\partial \theta_i}\}_{i=1}^{k+1}$ using $\{\frac{\partial B_{k+1}}{\partial \theta_i}\}_{i=1}^{k}$ via Equation (29), (34)
  /* Gradient */
  Calculate $\{\frac{\partial \mathbb{P}_k(j)}{\partial \theta_i}\}_{i=1}^{k}$ via Equations (11)-(18) in Section 2.5
end

S3 Prior distributions for EBDS models

S3.1 HIV dynamics in Odesa, Ukraine

We refer to the prior settings on the compound parameters from previous work (Vasylyeva et al. 2020), and try to roughly match their priors by adopting the following prior distributions on each of the rate parameters. Note that the sampling proportion was fixed to 0 before the first sampling date in their study, so we also set the sampling rate to 0 for the last two epochs for consistency.

bioRxiv preprint doi: https://doi.org/10.1101/2023.10.31.564882; this version posted November 2, 2023. The copyright holder for this preprint (which was not certified by peer review) is the author/funder, who has granted bioRxiv a license to display the preprint in perpetuity. It is made available under aCC-BY 4.0 International license.

Parameter	Prior	Role
λ	Lognormal (Mean = 0.85, SD = 1.0)	Birth rate
μ	Lognormal (Mean = −0.25, SD = 1.0)	Death rate
ψ	Lognormal (Mean = −9.0, SD = 0.50)	Serial sampling rate
t_{or}	Uniform (Lower = 19, Upper = 60)	Age of phylogeny

Table S1: Prior specifications for the EBDS model in HIV virus analysis

S3.2 Seasonal Influenza in New York State

We follow the same framework for setting the priors for the GMRF-based model as in Section S3.3. Similarly, the prior distribution for the constant death rate is acquired by estimating the credible range for the duration of the infectious period according to reports by Centers for Disease Control and Prevention (n.d.), with 95% confidence intervals encompassing 6 to 11 days. Comprehensive information regarding the specific prior distributions is shown in the following table:

Parameter	Prior	Role
λ_1^*	Normal (Mean = 3.08, SD = 1.17)	Log-scale birth rate at present
μ_k^*	Normal (Mean = 3.82, SD = 0.16)	Log-scale death rate for all epochs
ψ_1^*	Normal (Mean = −0.77, SD = 1.17)	Log-scale sampling rate at present
t_{or}	Normal (Mean = 12.5, SD = 15.0)	Age of phylogeny
α	Fixed to 2.0	Exponent of the MRF
ϕ	Gamma (Shape = 1.0, Scale = 1.0)	Transformed global scale of the MRF
ν_k	Fixed to 1.0	Local scale of MRF

Table S2: Prior specifications for the EBDS model in Influenza virus analysis

S3.3 Ebola epidemic in West Africa

We assume a constant death rate, μ for this data set, and we employ an empirical Bayes approach proposed by Magee et al. (2020) to set the prior on the first log-birth-rate and log-sampling-rate in our Bayesian bridge MRF models. The prior for the constant death rate is obtained from an estimation of the plausible duration of infectious period with 95% confidence intervals covering 8 to 40 days (Velásquez et al. 2015). The detailed prior distributions

bioRxiv preprint doi: https://doi.org/10.1101/2023.10.31.564882; this version posted November 2, 2023. The copyright holder for this preprint (which was not certified by peer review) is the author/funder, who has granted bioRxiv a license to display the preprint in perpetuity. It is made available under aCC-BY 4.0 International license.

803 can be found in the table below:

Parameter	Prior	Role
λ_1^*	Normal (Mean = 1.26, SD = 0.58)	Log-scale birth rate at present
μ_k^*	Normal (Mean = 3.02, SD = 0.41)	Log-scale death rate for all epochs
ψ_1^*	Normal (Mean = 1.27, SD = 0.58)	Log-scale sampling rate at present
t_{or}	Normal (Mean = 1.89, SD = 15.0)	Age of phylogeny
α	Fixed to 0.25	Exponent of the MRF
ϕ	Gamma (Shape = 1.0, Scale = 1.0)	Transformed global scale of the MRF
ν_k	Exponentially tilted stable distributions	Local scale of Bayesian bridge MRF
ξ	Fixed to 2.0	Slab width of Bayesian bridge MRF

Table S3: Prior specifications for the EBDS model in Ebola virus analysis

bioRxiv preprint doi: https://doi.org/10.1101/2023.10.31.564882; this version posted November 2, 2023. The copyright holder for this preprint (which was not certified by peer review) is the author/funder, who has granted bioRxiv a license to display the preprint in perpetuity. It is made available under aCC-BY 4.0 International license.

804 # S4 Inferred trajectories for birth/death/sampling rates

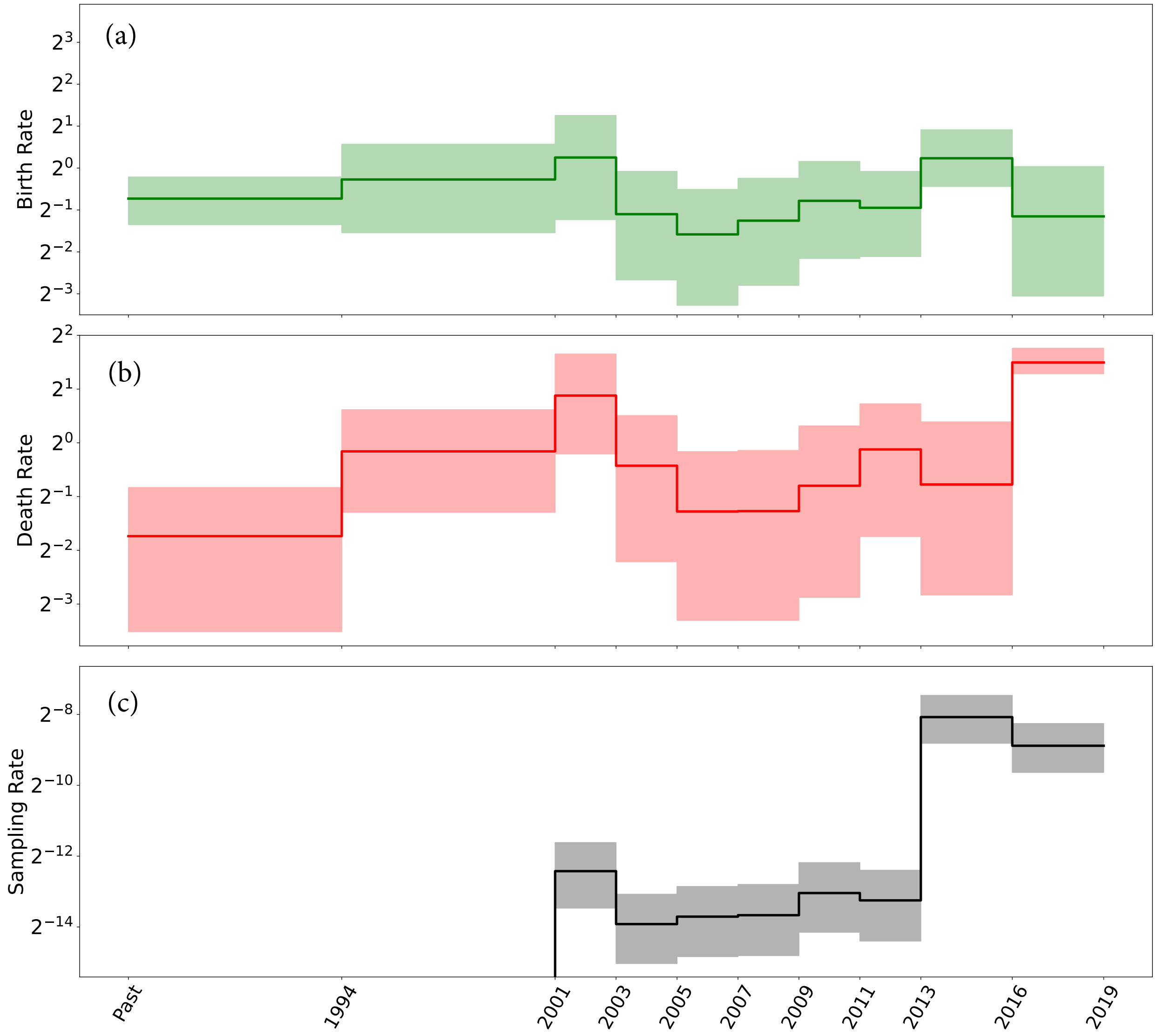

Figure S1: HIV virus: Median (solid line) and 95% credible intervals indicated by the shaded areas of the (a) birth rate, (b) death rate, and (c) sampling rate estimates through time.

bioRxiv preprint doi: https://doi.org/10.1101/2023.10.31.564882; this version posted November 2, 2023. The copyright holder for this preprint (which was not certified by peer review) is the author/funder, who has granted bioRxiv a license to display the preprint in perpetuity. It is made available under aCC-BY 4.0 International license.

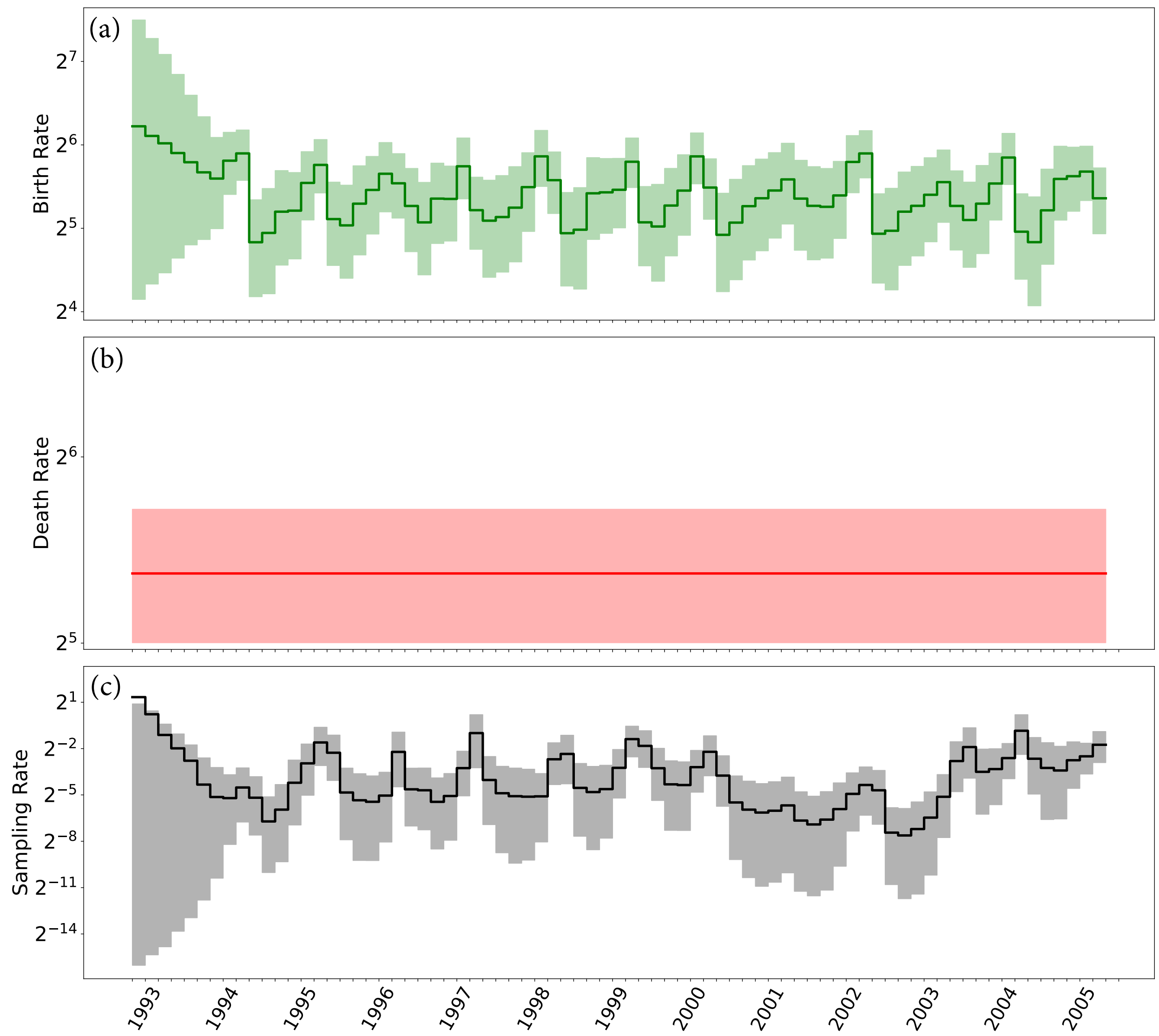

Figure S2: Influenza virus: Median (solid line) and 95% credible intervals indicated by the shaded areas of the (a) birth rate, (b) death rate, and (c) sampling rate estimates through time.

bioRxiv preprint doi: https://doi.org/10.1101/2023.10.31.564882; this version posted November 2, 2023. The copyright holder for this preprint (which was not certified by peer review) is the author/funder, who has granted bioRxiv a license to display the preprint in perpetuity. It is made available under aCC-BY 4.0 International license.

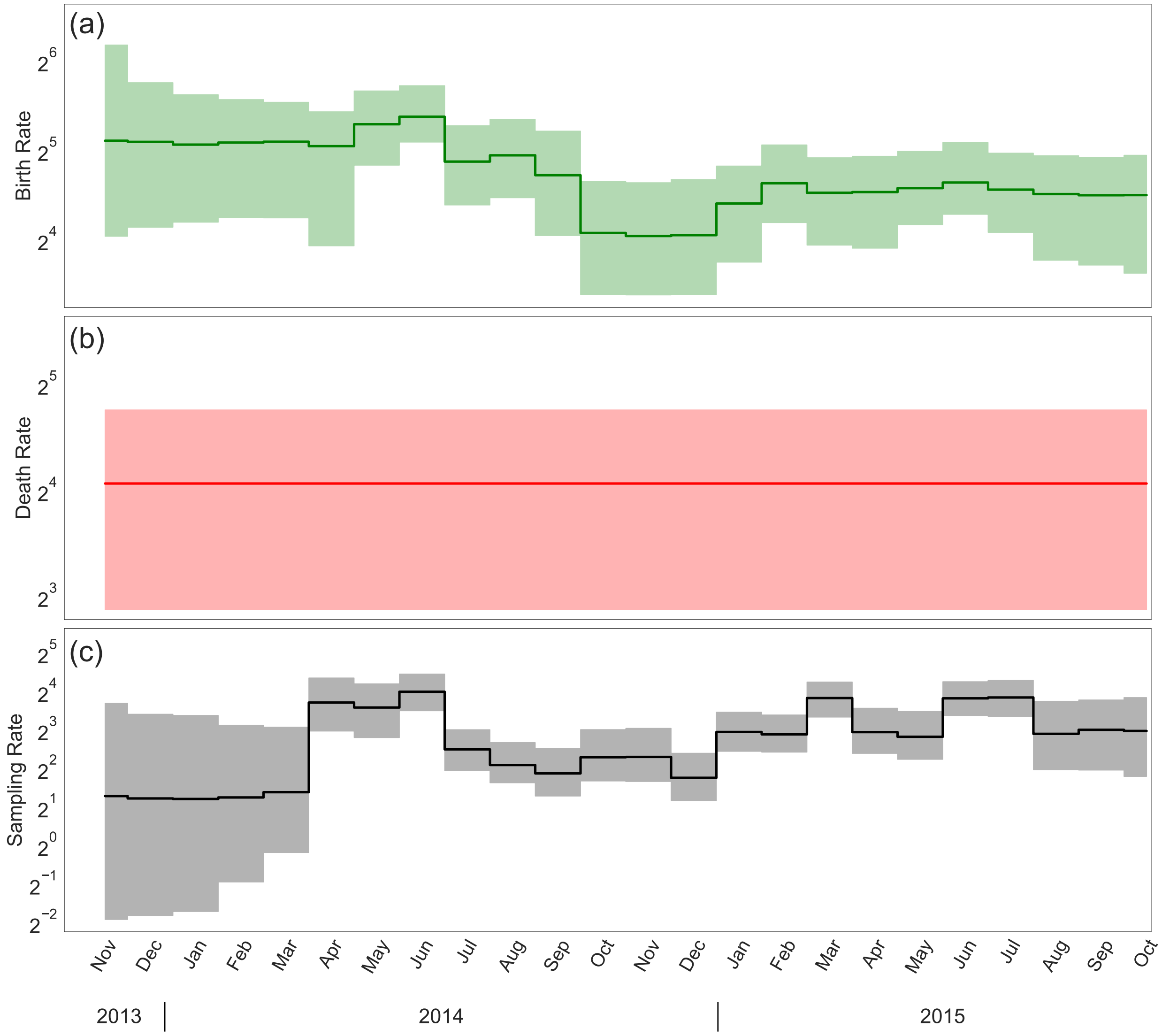

Figure S3: Ebola virus: Median (solid line) and 95% credible intervals indicated by the shaded areas of the (a) birth rate, (b) death rate, and (c) sampling rate estimates through time.

bioRxiv preprint doi: https://doi.org/10.1101/2023.10.31.564882; this version posted November 2, 2023. The copyright holder for this preprint (which was not certified by peer review) is the author/funder, who has granted bioRxiv a license to display the preprint in perpetuity. It is made available under aCC-BY 4.0 International license.

S5 Computational complexity of the nodewise likelihood

The computational complexity of evaluating node-based representations of the likelihood is much less explicit. First, we need to write out an equivalent expression for the likelihood of Equation 1 node-wise. It will be helpful to distinguish different types of samples. In particular, let us denote serially-sampled tips $\bar{\boldsymbol{u}}_\psi$ with a particular serially-sampled tip being $\bar{u}_{\psi i}$. With a slight abuse of notation, let us denote intensively-sampled tips $\bar{\boldsymbol{u}}_\rho$, with $\bar{\boldsymbol{u}}_{\rho i}$ denoting the *vector* of intensively-sampled tips at the ith intensive-sampling event. Then we can write

$$
\begin{aligned}
\mathbb{P}[\mathcal{T} \mid \boldsymbol{\lambda}, \boldsymbol{\mu}, \boldsymbol{\psi}, \boldsymbol{\rho}, \boldsymbol{r}, \boldsymbol{t}] = \log(q_{k(t_{or})}(t_{or})) + \left(\sum_{i=1}^{||\boldsymbol{v}||} \log(\lambda_{k(v_i)}) + \log(q_{k(v_i)}(v_i)) \right) + \\
\left(\sum_{i=1}^{||\bar{\boldsymbol{u}}_\psi||} \log(\psi_{k(\bar{u}_{\psi i})}) + \log(r_{k(\bar{u}_{\psi i})} + (1 - r_{k(\bar{u}_{\psi i})}) p_{k(\bar{u}_{\psi i})})) - \log(q_{k(\bar{u}_{\psi i})}(\bar{u}_{\psi i})) \right) + \\
\left(\sum_{i=1}^{K} ||\bar{\boldsymbol{u}}_{\rho i}|| + \log(\rho_i) + (L(t_{i-1}) - ||\bar{\boldsymbol{u}}_{\rho i}||) \log((1-\rho_i) q_{i-1}(t_{i-1})) + \right. \\
\left. + ||\tilde{\boldsymbol{u}}_{\rho i}|| \log(1 - r_i) q_{i-1}(t_{i-1}) ||\bar{\boldsymbol{u}}_{\rho i}|| \log(r_i + (1 - r_i) p_{i-1}(t_{i-1})) \right)
\end{aligned}
\tag{36}
$$

The complexity here is not immediately apparent for a number of reasons. For one, the complexity appears to depend on the relative proportion of samples of different types, which affects the number of values of $p_k(t)$ and $q_k(t)$ which must be computed. Importantly, the complexity of computing those $p_k(t)$ and $q_k(t)$ is not immediately apparent either, and that these costs are somewhat hard to disentangle, as $p_k(t_i)$ builds recursively on $p_{k-1}(t_i)$ and $q_k(t)$ depends on $p_k(t)$.

bioRxiv preprint doi: https://doi.org/10.1101/2023.10.31.564882; this version posted November 2, 2023. The copyright holder for this preprint (which was not certified by peer review) is the author/funder, who has granted bioRxiv a license to display the preprint in perpetuity. It is made available under aCC-BY 4.0 International license.

S5.1 Node lookups

Regardless of such ambiguities, all nodes in the tree require an interval lookup. For births, the lookup is required to find the correct λ_k term to use. For samples, the lookup is either to find the appropriate sampling rate, for serial samples, or to determine to which intensive-sampling event a sample belongs, for intensive samples. The time requirement here depends on the algorithm, for a binary search it is $\mathcal{O}(\log(K))$, making the total lookup cost $\mathcal{O}(N \log(K))$.

S5.2 How many computations of $q_k(t)$ are required?

In the worst, but most common, case, there are no intensive-sampling events and $q_k(t)$ must be computed for the times of all samples, all births, and all epoch times (note that even when ρ_i is 0, there is a term $L(t_i) \log(q_{i-1}(t_i))$ which must be computed in the final summation). In the best case, all samples are at intensive-sampling events, and $q_k(t)$ only needs to be computed for the times of all births and all epoch times. These are both $\mathcal{O}(N + K)$, though there is a factor of two's worth of variation in front of the N depending on which side of this spectrum a tree falls in. Calling the cost of computing $q_k(t)$ Q, this makes the contribution to the complexity here $\mathcal{O}(Q(N + K))$.

S5.3 How many computations of $p_k(t)$ are required?

The likelihood contains a number of explicit computations of $p_k(t)$ in the terms pertaining to (both serially- and intensively-)sampled tips. When all samples are serial samples, there are $\mathcal{O}(N)$ direct computations of $p_k(t)$, while when all samples are intensive samples, there are $\mathcal{O}(K)$. Taking the cost of computing $p_k(t)$ to be P, the addition to the cost here is between $\mathcal{O}(PN)$ and $\mathcal{O}(PK)$.

bioRxiv preprint doi: https://doi.org/10.1101/2023.10.31.564882; this version posted November 2, 2023. The copyright holder for this preprint (which was not certified by peer review) is the author/funder, who has granted bioRxiv a license to display the preprint in perpetuity. It is made available under aCC-BY 4.0 International license.

S5.4 What is the cost of computing $p_k(t)$ and $q_k(t)$?

We have thus far shown that the cost of computing the nodewise likelihood appears to be between $\mathcal{O}(N\log(K) + Q(N+K) + PN)$ and $\mathcal{O}(N\log(K) + Q(N+K) + PK)$. But this is not particularly revealing without considering P and Q.

While $q_k(t)$ depends on $p_{l:l<k}(t)$ through $\mathbf{A}$ and $\mathbf{B}$, once A_k and B_k have been computed, let us assume (as we did when evaluating the cost of the interval-wise likelihood) that the cost of $q_k(t)$ is $\mathcal{O}(1)$. In other words, let us assume that $\mathcal{O}(Q(N+K)) = \mathcal{O}(P(N+K))$. This makes the implied cost of the nodewise likelihood between $\mathcal{O}(N\log(K) + P(N+K) + PN)$ and $\mathcal{O}(N\log(K) + P(N+K) + PK)$, which both simplify to $\mathcal{O}(N\log(K) + P(N+K))$. Naïvely, we might choose to compute $p_k(t)$ recursively every time we need it, which is $\mathcal{O}(K^2)$. In this case, the implied cost of the nodewise likelihood is $\mathcal{O}(N\log(K) + NK + K^2))$.

S5.5 Precomputing $\mathbf{A}$ and $\mathbf{B}$

One can instead choose to pre-compute A_k, B_k, as once these are computed the cost to compute $p_k(t)$ and $q_k(t)$ becomes $\mathcal{O}(1)$. Working backwards from the present allows re-computation to be avoided. As we did when we approximated the cost of the interval-wise likelihood, we will take the cost of the update (computing (A_k, B_k) from (A_{k-1}, B_{k-1})) to be $\mathcal{O}(1)$. Thus, the cost of the precomputation is $\mathcal{O}(K)$. This puts the implied cost of computing the nodewise likelihood between $\mathcal{O}(N\log(K) + N + K)$.

S5.6 Counting lineages at epoch times

Regardless of whether the model includes intensive-sampling (that is, regardless of whether $\boldsymbol{\rho} = 0$), one must compute $L(t_i)$ for all epoch times. This can be solved essentially the same way as the subintervals are obtained, at a cost of $\mathcal{O}(N + N\log(N))$. Alternately, it can be obtained by counting the number of births and sampled tips older (or younger) than each epoch time, at a cost of $\mathcal{O}(KN)$. This makes the lower end of the computational cost once

bioRxiv preprint doi: https://doi.org/10.1101/2023.10.31.564882; this version posted November 2, 2023. The copyright holder for this preprint (which was not certified by peer review) is the author/funder, who has granted bioRxiv a license to display the preprint in perpetuity. It is made available under aCC-BY 4.0 International license.

again a range, from $\mathcal{O}(NK + N\log(K) + N + K)$ to $\mathcal{O}(N\log(K) + N\log(N) + N + K)$.

In practice, the constants in front of all the sorting and node-lookup terms appear to be so small as to be unnoticeable in real-world computation. We demonstrate this in our timing experiments in the next section. Thus, for all practical purposes, the likelihood appears to be $\mathcal{O}(N + K)$ regardless of representation, as long as one avoids recursive computation of $p_k(t)$.

bioRxiv preprint doi: https://doi.org/10.1101/2023.10.31.564882; this version posted November 2, 2023. The copyright holder for this preprint (which was not certified by peer review) is the author/funder, who has granted bioRxiv a license to display the preprint in perpetuity. It is made available under aCC-BY 4.0 International license.

S6 Timing Experiments

With the reformulation of the likelihood and derivation of the analytical gradients, our method notably gains in speed, as we highlight in this section. For a comprehensive assessment, we compare our approach with four other specialized packages for EBDS model inference concerning likelihood calculations. These include the BDSKY (Stadler et al. 2013) package within BEAST2 (Bouckaert et al. 2019), TreePar (Stadler et al. 2013) package in R (R Core Team 2021) and RevBayes (Höhna et al. 2016). Furthermore, we present a benchmark comparing the gradient calculation efficiency of automatic differentiation implemented in VBSKY (Ki & Terhorst 2022)package using JAX library (Bradbury et al. 2018) isolated from the variational inference procedure against our algorithm based analytical gradients implemented in BEAST.

To assess the scalability of the aforementioned methods in terms of likelihood/gradient calculation, we simulated a set of trees under the EBDS model with increasing number of tips. To investigate the scalability of different methods wrt the number of sequences, we fix the number of epochs to 5 for both likelihood and gradient calculation.

Regarding scalability with respect to the number of epochs, we adjust the model by progressively increasing the number of epochs. To keep other variables constant, we maintain the tree topology and set the number of tips at 12 (in scenarios where $K >> N$, this allows us to negate the effect of N in $\mathcal{O}(N+K)$) for likelihood computation. For gradient calculations, we set the number of tips to 8198 (to minimize the impact of K^2 in $\mathcal{O}(NK+K^2)$).

For methods that employ just-in-time (JIT) compilation, including BEAST, BEAST2 and VBSKY, we run a short MCMC chain or variational inference algorithm to compute likelihood or gradient across 100,000 iterations and take the average run time.

bioRxiv preprint doi: https://doi.org/10.1101/2023.10.31.564882; this version posted November 2, 2023. The copyright holder for this preprint (which was not certified by peer review) is the author/funder, who has granted bioRxiv a license to display the preprint in perpetuity. It is made available under aCC-BY 4.0 International license.

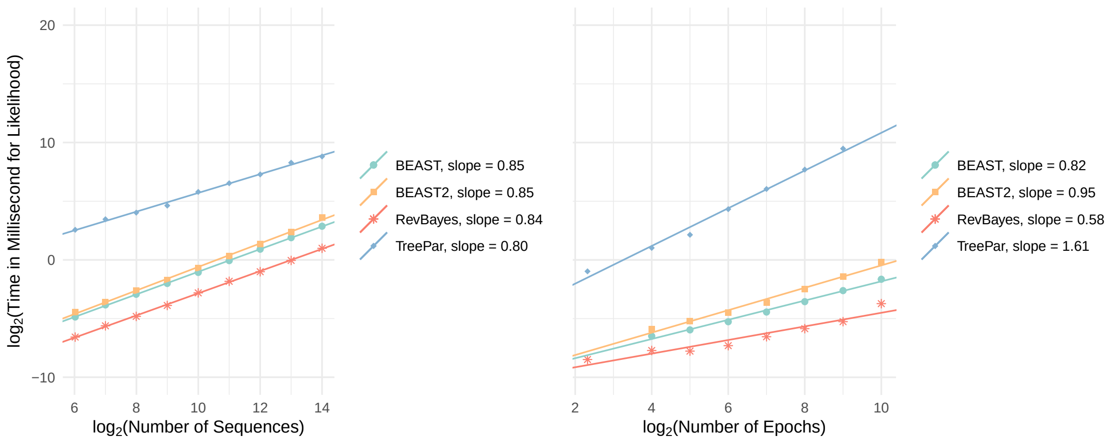

Figure S4: Speed of implementations for the likelihood calculations of increasing number of sequences (left plot) or number of epochs (right plot) for EBDS model. Note the time and number of sequences/epochs are laid out according to a logarithmic scale with base 2.

In our analysis, we observe that for likelihood computations, the implementations in BEAST, BEAST2, and RevBayes offer similar speed performance when adjusting both the number of sequences and epochs. In contrast, the TreePar package consistently lags, being several hundred times slower than its counterparts across all tested scenarios. It is also the sole implementation that exhibits a quadratic scaling with the number of epochs. The algorithms of BEAST, BEAST2, and RevBayes seem to demonstrate approximately linear scaling relative to both tree size and model epochs. It's worth noting that RevBayes delivers the quickest calculation speed, which might be attributed to the inherent speed advantages of precompiled codes, particularly for quick likelihood calculations in our context. Result for TreePar with epochs exceeding than 512 is not not included as TreePar fail to process such large models.

bioRxiv preprint doi: https://doi.org/10.1101/2023.10.31.564882; this version posted November 2, 2023. The copyright holder for this preprint (which was not certified by peer review) is the author/funder, who has granted bioRxiv a license to display the preprint in perpetuity. It is made available under aCC-BY 4.0 International license.

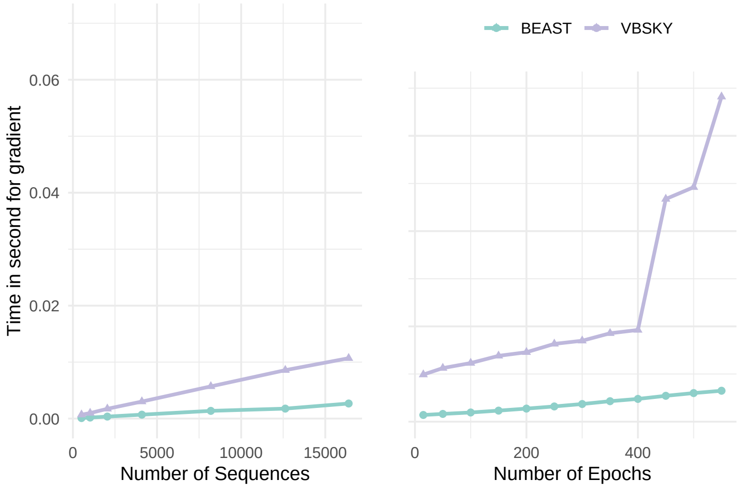

Figure S5: Speed of implementations for and gradient calculations of increasing number of sequences (left plot) or number of epochs (right plot) for EBDS model.

In terms of gradient calculations, our analytical gradients deployed within BEAST is remarkably faster than VBSKY approach using automatic differentiation. The gradient computation scales approximately linearly with the number of sequences for both BEAST and VBSKY. However, wrt the number of epochs, the scaling remains linear for BEAST but seems quadratic for VBSKY. We further confirm that the runtime slowness exhibited in VBSKY is not due to memory issues or JIT compilation difficulty. Therefore, our analysis demonstrates that analytically calculating the gradients of the EBDS likelihood is critical for improving the running time of gradient based methods.
